# TLR9 signaling through NF-κB/RELA and STAT3 promotes tumor-propagating potential of prostate cancer cells

**DOI:** 10.18632/oncotarget.4029

**Published:** 2015-05-22

**Authors:** Dayson Moreira, Qifang Zhang, Dewan Md S. Hossain, Sergey Nechaev, Haiqing Li, Claudia M. Kowolik, Massimo D'Apuzzo, Stephen Forman, Jeremy Jones, Sumanta K. Pal, Marcin Kortylewski

**Affiliations:** ^1^ Department of Cancer Immunotherapeutics & Tumor Immunology, Beckman Research Institute at City of Hope, Duarte, CA 91010, USA; ^2^ Bioinformatics Core Facility, Beckman Research Institute at City of Hope, Duarte, CA 91010, USA; ^3^ Department of Molecular Medicine, Beckman Research Institute at City of Hope, Duarte, CA 91010, USA; ^4^ Department of Pathology, Beckman Research Institute at City of Hope, Duarte, CA 91010, USA; ^5^ Department of Hematologic Malignancies, Beckman Research Institute at City of Hope, Duarte, CA 91010, USA; ^6^ Department of Cell Biology, Beckman Research Institute at City of Hope, Duarte, CA 91010, USA; ^7^ Department of Medical Oncology, Beckman Research Institute at City of Hope, Duarte, CA 91010, USA; ^8^ Irell & Manella Graduate School of Biological Sciences, Beckman Research Institute at City of Hope, Duarte, CA 91010, USA

**Keywords:** TLR9, prostate cancer, RELA, STAT3, tumor-propagating cells

## Abstract

Prostate cancer progression was associated with tumorigenic signaling activated by proinflammatory mediators. However, the etiology of these events remains elusive. Here, we demonstrate that triggering of the innate immune receptor, Toll-like Receptor 9 (TLR9), in androgen-independent prostate cancer cells initiates signaling cascade leading to increased tumor growth and progression. Using limited dilution/serial transplantation experiments, we show that TLR9 is essential for prostate cancer cells' potential to propagate and self-renew *in vivo*. Furthermore, low expression or silencing of TLR9 limits the clonogenic potential and mesenchymal stem cell-like properties of LNCaP- and PC3-derived prostate cancer cell variants. Genome-wide transcriptional analysis of prostate cancer cells isolated from xenotransplanted TLR9-positive and -negative tumors revealed a unique gene expression signature, with prominent upregulation of inflammation- and stem cell-related markers. TLR9 signaling orchestrated expression of critical stem cell-related genes such as *NKX3.1, KLF-4, BMI-1* and *COL1A1*, at both mRNA and protein levels. Our further analysis identified that TLR9-induced NF-κB/RELA and STAT3 transcription factors co-regulated *NKX3.1* and *KLF4* gene expression by directly binding to both promoters. Finally, we demonstrated the feasibility of using TLR9-targeted siRNA delivery to block RELA- and STAT3-dependent prostate cancer cell self-renewal *in vivo*. The intratumoral administration of CpG-*RELA*siRNA or CpG-*STAT3*siRNA but not control conjugates inhibited growth of established prostate tumors and reduced clonogenic potential of cancer cells. Overcoming cancer cell self-renewal and tumor-propagating potential by targeted inhibition of TLR9 signaling can provide therapeutic strategy for late-stage prostate cancer patients.

## INTRODUCTION

In developed countries, prostate cancer is the second leading cause of male cancer-related deaths with lack of effective therapies for late-stage cancer patients [1]. The initiation, progression and metastasis of prostate cancers is thought to be associated with chronic or recurrent inflammation as underscored by numerous studies and epidemiological evidence [2, 3].

Cellular reactions to infection, tissue stress and injury involve activation of Toll-like receptors (TLRs) in various hematopoietic cells [4]. More recent studies found that TLRs, such as TLR9, are expressed by solid tumors including prostate cancers [5]. Cancer cells can also upregulate TLR9 in response to genotoxic stress caused by irradiation or chemotherapy [6]. Rather than becoming immunogenic, TLR9^+^ prostate cancers are reportedly less differentiated, more aggressive and prone to reoccur [7, 8]. TLR9 recognizes DNA molecules harboring unmethylated CpG motifs, typical for bacterial DNA [4]. Recent studies documented that mitochondrial DNA (mtDNA) released as a result of sterile tissue injury activates TLR9 causing pathologic inflammatory responses [9, 10]. Downstream TLR9 signaling involves NF-κB transcription factor, which regulate expression of proinflammatory and survival mediators [4]. In immune cells, TLR9 signaling is tightly controlled at multiple levels by the signal transducer and activator of transcription 3 (STAT3) [11, 12]. STAT3 is an essential negative feedback inhibitor for TLR9 signaling which is activated by NF-κB-induced cytokines such as IL-6 or IL-10 [9, 13, 14]. Little is known about downstream effects of TLR9 signaling in human cancer cells. However, there is compelling evidence on the role of NF-κB and STAT3 in prostate cancer cell proliferation, survival and androgen-independence. [15–18]

In the current study, we investigated whether inflammatory TLR9 signaling in prostate cancer cells provides a set of molecular targets driving tumor aggressiveness and tumor-propagating potential. These studies provide insights into tumorigenic role of inflammation in advanced prostate tumors.

## RESULTS

### TLR9 promotes prostate cancer cell engraftment and progression *in vivo*

**Figure 1:**
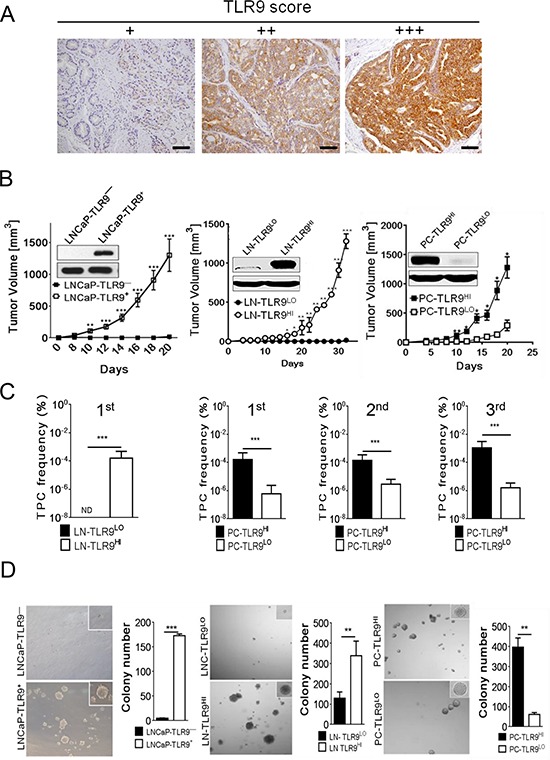
Higher frequency of self-renewing tumor-propagating cells (TPC) in TLR9 + prostate tumors **A.** The advanced human prostate cancers express TLR9 as assessed by immunohistochemical staining and pathological evaluation on sections from primary prostate cancers; scale bars = 10 μm. TLR9 levels were assessed within foci of carcinoma representative of the final Gleason score and the cytoplasmic staining was scored as: negative, no staining; +, staining in < 20% of carcinoma cells; ++, weak to moderate staining in > 20% of carcinoma cells; +++, strong/diffuse staining in > 20% of carcinoma cells. **B.** TLR9 expression promotes engraftment and growth of prostate cancer xenotransplants. Human TLR9 was either expressed in LNCaP and LNCaP-S17 cancer cells (with low basal levels of TLR9) or silenced in PC3 cells (with high basal levels of TLR9) using lentiviral systems. The protein levels of TLR9 were assessed using Western blotting; β-actin was used as a loading control (inlays). The immunodeficient NSG mice were injected subcutaneously using 5 × 10^6^ LNCaP- (left panel), LNCaP-S17- (middle panel), or PC3-derived cell variants (right panel). Tumor growth was measured at the indicated times; means ± SEM (*n* = 5). The results represent three independent experiments. **C.** The primary, secondary and tertiary TPC frequencies were measured in LN-TLR9^LO^ and LN-TLR9^HI^ or in PC-TLR9^LO^ and PC-TLR9^HI^ tumors using limiting dilution analysis *in vivo*; shown are means and 95% CI. **D.** Prostate cancer cells expressing TLR9 have augmented clonogenic potential. Cancer cells freshly isolated from xenotransplanted LNCaP (left), LNCaP-S17 (middle panel) or PC3 (right panel) variants were grown in 3D cultures to form colonies. Shown are the representative images and numbers of spheroid colonies; means ± SEM (*n* = 5).

Previous studies reported expression of the innate immune receptor TLR9 in human prostate cancer cells [5, 7, 8]. We verified these findings by histopathology on 48 primary prostate cancer specimens. Although TLR9 expression showed inter- and intratumoral variation, all samples showed at least low level of cytoplasmic TLR9 (Figure 1A). The staining intensity correlated with increased Gleason grade for the majority of samples (Supplementary Table S1). To assess whether TLR9 contributes to prostate cancer progression, we selected three prostate cancer models: parental LNCaP cells, LNCaP-S17 cells stably expressing IL-6 and PC3-luc cells as representing less or more advanced stages in the progression to androgen-independence, respectively [19]. The TLR9 protein levels were undetectable in LNCaP, low in LNCaP-S17 and high in PC3 cells (Figure 1B, inlays). To study the role of TLR9, we stably transduced the LNCaP and LNCaP-S17 cells using lentiviruses encoding either human *TLR9* cDNA (LNCaP-TLR9^+^ and LN-TLR9^HI^) or mock vector (LNCaP-TLR9^−^ and LN-TLR9^LO^); meanwhile the PC3 cells were transduced with either *TLR9* shRNA (PC-TLR9^LO^) or non-silencing control vector (PC-TLR9^HI^) (Figure 1B, inlays). In both LNCaP and PC3 cell variants higher levels of TLR9 expression and activation (by CpG ODN stimulation) correlated with increased mRNA and protein levels of IL-6, an important STAT3 activator and a contributor to prostate cancer progression [19]. As expected, these effects were blunted in LNCaP-S17 cells overexpressing IL-6 (Supplementary Figure S1). The *in vitro* proliferation of these cancer cells variants did not significantly change (Supplementary Figure S2AB). To evaluate TLR9 effect on prostate tumor progression, we injected LNCaP, LNCaP-S17 and PC3 cell variants subcutaneously into immunodeficient NSG mice. Both LNCaP-TLR9^+^ and LN-TLR9^HI^ cells formed progressively growing tumors in contrast to poorly tumorigenic LNCaP-TLR9^−^ and LN-TLR9^LO^ cells (Figure 1B, left/middle). Although the PC-TLR9^LO^ tumors became palpable within two weeks, their growth was strongly delayed compared to PC-TLR9^HI^ tumors (Figure 1B, right). Overall, in all tested prostate cancer models, high TLR9 expression correlated with tumor engraftment and growth.

### TLR9 increases frequency of prostate cancer stem-like cells with self-renewal properties

Prior studies linked increased tumorigenicity to a population of prostate cancer stem cells which enable serial tumor transplantation [20, 21]. To assess frequency of tumor-propagating cell (TPC) and their self renewal potential in variants of LNCaP-S17 and PC3 cells, we used limited-dilution/clonal tumor-initiation assays [21]. The LN-TLR9^LO^ tumors showed limited and delayed engraftment in NSG mice, thus preventing us from the TPC assessment within the timeframe of our analysis (Figure 1C). In contrast, the TPC frequency in LN-TLR9^HI^ tumors was high and comparable to TPC numbers in the PC-TLR9^HI^ model (Figure 1C, first panel). The silencing of TLR9 in PC-TLR9^LO^ cells resulted in ~200-fold reduction in the TPC frequency (Figure 1C, second panel) which corresponded to the previously observed delayed PC-TLR9^LO^ tumor engraftment (Figure 1B, right). To confirm the enhanced self-renewal properties of PC-TLR9^HI^ cells, we transplanted tumor cells using limited dilution, from primary into secondary and then tertiary recipients (Figure 1C, three right panels). The significant reduction in the TPC frequency was consistent throughout serial transplantations of PC3 variants. Differences in TPC frequencies between prostate cancer variants could reflect changes in putative stem-like/progenitor cell populations [20, 21]. We used standard colony/sphere formation assays to verify whether TLR9 expression affects clonogenic potential of prostate cancer cells. Within 7–14 days, both LNCaP-TLR9^+^ and LN-TLR9^HI^ cells formed prostatospheroids while LNCaP-TLR9^−^ and LN-TLR9^LO^ cells were only loosely clustered (Figure 1D, left/middle). Both variants of PC3 cell created large and regular prostatospheres, however the number of colonies was reduced 8-fold after TLR9 silencing (Figure 1D, right).

Prostate cancer cells often demonstrate bone marrow mesenchymal stem cells' features [22]. Under *in vitro* stimulation both LNCaP and PC3 cancer cells differentiate into either osteoblast- or adipocyte-like cells [22]. Thus, we tested whether upregulation of TLR9 levels will stimulate such properties of prostate cancer cells. Depending on culture conditions, both LN-TLR9^HI^ and PC-TLR9^HI^ cells differentiated into adipocyte- or osteoblast-like cells while prostate cancer cells with low levels of TLR9 failed to differentiate (Supplementary Figure S3). Together with our prior results, these observations support the notion that TLR9 expression in prostate cancer cells promotes tumor-propagating and stem cell-like phenotype, which is likely responsible for enhanced aggressiveness.

### TLR9 orchestrates expression of tumorigenic and stem cell-related genes in prostate cancer cells

**Figure 2:**
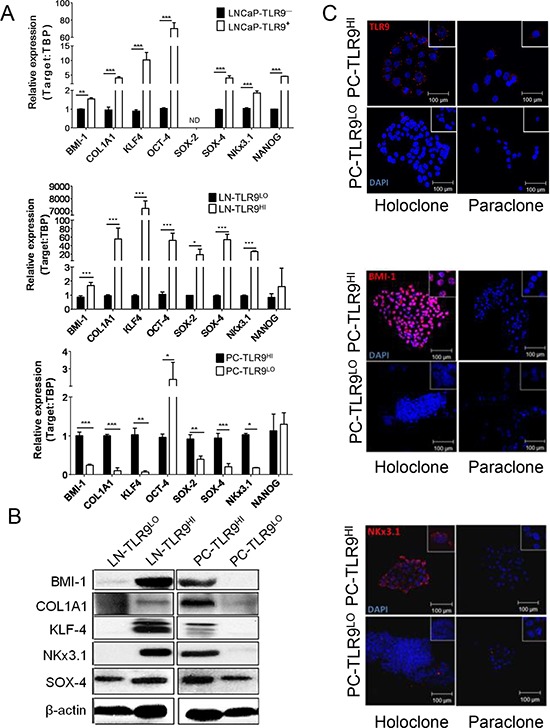
TLR9 orchestrates expression of prostate cancer stem cell-related genes **A.** The mRNA expression of *BMI-1, COL1A1, KLF4, OCT-4, SOX-2, SOX-4*, *NKX3.1* and *NANOG* was assessed by qPCR in RNA samples isolated from LNCaP cells (top) or xenotransplanted LNCaP-S17 (middle) and PC3 (bottom) tumor variants; means ± SEM (*n* = 3). **B.** Protein levels of the selected stem cell-related factors as indicated were assessed using Western blotting; β-actin was used as a loading control. **C.** Intracellular localization of TLR9 (top), BMI-1 (middle) and NKX3.1 (bottom) shown in red with DAPI for staining nuclei (blue) in holo-/paraclones formed by PC-TLR9^LO^ or PC-TLR9^HI^ cells *in vitro* and analyzed using immunofluorescent microscopy; scale bar = 100 μm.

TLR9 regulates expression of genes critical for function of non-malignant immune cells [4] but the TLR9 downstream gene targets in cancer cells are mostly unknown. We employed whole transcriptome profiling to dissect the TLR9's role in prostate cancer cells (Supplementary Figure S4). Altogether, our results revealed the potential link between TLR9 signaling and gene networks involved in stem cell maintenance and renewal. For validating this prediction, we selected a set of prostate cancer stem cell-specific genes: *BMI-1*, *NKX3.1* and *SOX-4*; mesenchymal stem cell-related *COL1A1*; and critical regulators of pluripotent embryonic stem cells (ESC): *KLF-4*, *SOX-2*, *OCT-4* and *NANOG*. [23–26] The qPCR analysis of total RNA samples isolated from cultured LNCaP-TLR9 variants or *in vivo* grown LN-TLR9^HI^ and LN-TLR9^LO^ tumors showed the significant upregulation of majority of the tested stem cell-related genes after TLR9 expression (Figure 2A, top/middle). Correspondingly, the silencing of TLR9 in PC3 cells (PC-TLR9^LO^) significantly downregulated of the majority (6/8) of tested genes, except for canonical ESC factors *NANOG* and *OCT4* (Figure 2A, bottom; Supplementary Figure S5A–B). In addition, TLR9 expression correlated with the protein levels of NKX3.1, KLF-4, BMI-1 and COL1A1 in both LNCaP-S17 and PC3 cells (Figure 2B). When seeded in 2D culture at clonal density, PC3 cells form two types of keratinocyte-like colonies: the dispersed paraclones and the regularly-shaped holoclones. The latter type reportedly harbors stem-like cells with self-renewal potential [27]. Our initial studies indicated that TLR9 expression correlated with the number of holoclones formed by LNCaP, LNCaP-S17 and PC3 cell variants (Supplementary Figure S6). Thus, we combined immunofluorescent staining with clonal cell-dilution assays to evaluate TLR9, NKX3.1 and BMI-1 expression in PC3 cell variants. In fact, PC-TLR9^HI^ cell holoclones showed higher than paraclones levels of TLR9 (Figure 2C, top). Furthermore, we observed that BMI-1 and NKX3.1 were expressed exclusively in PC-TLR9^HI^ holoclones (Figure 2C, middle and bottom). These results suggested that TLR9 drives the transcriptional program, which promotes the stem cell-like phenotype in prostate cancer cells.

### *NF-κB* and *STAT3* cooperate to mediate TLR9-induced effects in prostate cancer cells

**Figure 3:**
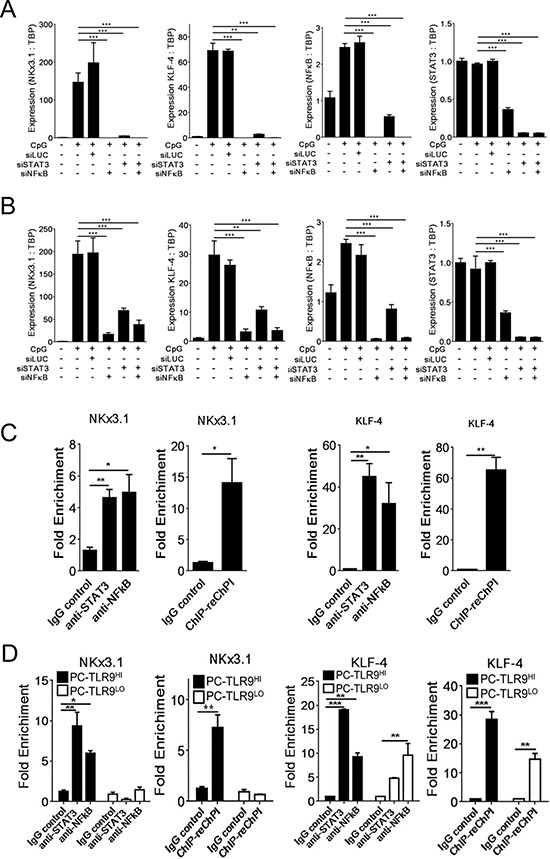
NF-κB and STAT3 co-regulate expression of NKX3.1 and KLF-4 prostate cancer stem cell-related genes downstream from TLR9 **A, B.** Both NF-κB and STAT3 are required for TLR9-dependent expression of *NKX3.1* and *KLF-4* genes. 10^6^ LNCaP-TLR9^+^ (A) or LN-TLR9^HI^ cells (B) were transfected using Lipofectamine 2000 (Invitrogen) and siRNAs specific to *NF-κB/RELA*, *STAT3* or *Luciferase* as a negative control (40 nm/L). After 24 h, cells were treated using CpG ODN (1 μg/mL) for another 24 h or left untreated. The mRNA levels of *NKX3.1* and *KLF4* as well as *NF-κB/RELA* and *STAT3* for verification of target gene silencing were assessed by qPCR and normalized to *TBP* expression. Shown are results from two independent experiments; means ± SEM. **C.** TLR9 activation in prostate cancer cells induces binding of both NF-κB/RELA and STAT3 to promoters of stem cell-related genes. The LN-TLR9^HI^ cells were stimulated using CpG as above. NF-κB/RELA and STAT3 binding to *NKX3.1* (left two graphs) and *KLF4* (right two graphs) promoters was evaluated using ChIP assays with qPCR analyses. In addition, a portion of the anti-STAT3 immunoprecipitate was subjected to a second round of immunoprecipitation using NF-κB/RELA antibody (re-Chip) as indicated. **D.** TLR9 silencing reduces NF-κB/RELA and STAT3 recruitment to *NKX3.1* and *KLF4* promoters in PC3 cells. ChIP (left) and Re-ChIP assays (right) were performed using PC-TLR9^LO^ and PC-TLR9^HI^ cells treated using CpG ODN for 1 h. *NKX3.1* and *KLF4* promoter-binding activities of NF-κB/RELA and STAT3 were assessed as fold enrichment in two independent experiments; mean ± SEM (*n* = 4).

The IPA analysis of our RNAseq data suggested NF-κB/RELA and STAT3 as top mediators of TLR9-dependent gene expression in prostate cancer cells (Supplementary Figure S5C). To elucidate TLR9-mediated transcriptional regulation, we focused on two key prostate cancer stem cell-related genes: *NKX3.1* and *KLF-4*. The CpG-induced upregulation of *NKX3.1* and *KLF4* was inhibited by the silencing of either *NF-κB/RELA* or *STAT3* in both LNCaP-TLR9^+^ (Figure 3A) and LN-TRL9^HI^ cells (Figure 3B). Next, we used the chromatin immunopreciptation (ChIP) and qPCR assays to test whether TLR9 stimulates binding of NF-κB/RELA and/or STAT3 to *NKx3.1* or *KFL-4* promoter regions in LN-TLR9^HI^ cells. In fact, within an hour after TLR9 activation both NF-κB/RELA and STAT3 were recruited to *NKX3.1* and *KFL-4* promoters (Figure 3C). NF-κB/RELA and STAT3 potentially partner in transcriptional regulation of tumorigenic genes in cancer cells [16]. We used Re-ChIP assays to assess whether these TFs bind to the same DNA regulatory elements in *NKX3.1* and *KFL-4* promoters. The sequential immunoprecipitation using STAT3- and then NF-κB/RELA-specific antibodies yielded promoter regions from both *NKX3.1* and *KLF-4* (Figure 3C). We further evaluated these results using ChIP/Re-ChIP assays in PC3-TLR9^LO^ and PC3-TLR9^HI^ cells. As expected, CpG stimulation induced binding of NF-κB/RELA together with STAT3 to the *NKX3.1* promoter in PC3-TLR9^HI^ but not in PC3-TLR9^LO^ cells (Figure 3D, left). NF-κB/RELA and STAT3 similarly collaborate for the transcriptional regulation of *KLF-4* in PC3-TLR9^HI^ cells (Figure 3D, right). TLR9 silencing reduced STAT3 binding to *KLF-4* promoter, although it did not affect NF-κB/RELA. It is likely that the interaction with STAT3 supports but is not required for NF-κB binding to *KLF-4* promoter. Taken together, our results suggest that NF-κB/RELA and STAT3 cooperate to various extents in orchestrating expression of TLR9-induced prostate cancer stem cell-related genes.

### Targeted inhibition of NF-κB and STAT3 signaling in TLR9-positive tumor-propagating cells inhibits growth of human prostate cancers *in vivo*

**Figure 4:**
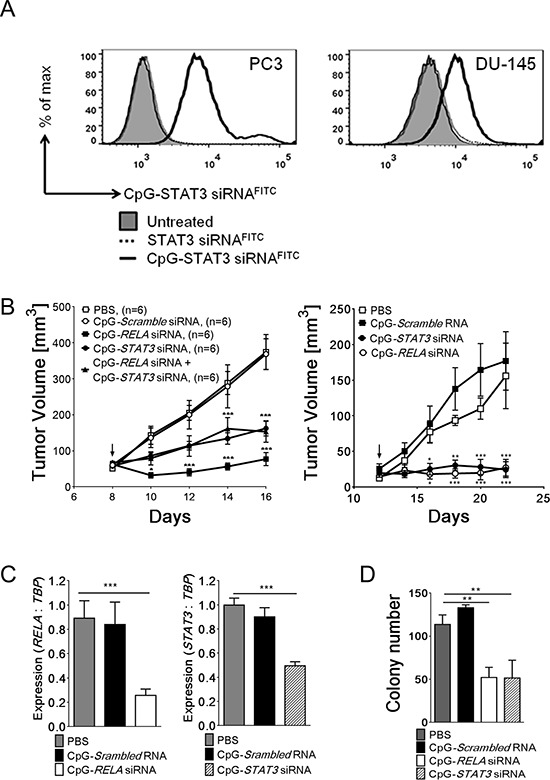
TLR9-targeted silencing of RELA or STAT3 inhibits growth and clonogenic potential of prostate tumors **A.** Internalization of naked CpG-siRNA conjugates by PC3-luc and DU145 prostate cancer cells in the absence of transfection reagents. Cells were incubated with fluorescently-labeled CpG-*STAT3*siRNA^FITC^ or unconjugated *STAT3*siRNA^FITC^ (500 nM) for 1 h and the level of uptake was analyzed using flow cytometry. **B.** CpG-mediated silencing of *RELA* and/or *STAT3* inhibits growth of PC3-luc (left) and DU145 (right) prostate tumors in NSG mice. After tumors well established (as shown by arrows), the established PC3-luc tumors were treated using IT injections of the indicated CpG-siRNA conjugates, CpG-scrambled RNA control (5 mg/kg) or PBS every other day while measuring tumor volumes. **C.** The silencing effect of CpG-siRNA conjugates in PC3 cells were verified using qPCR to measure *RELA* (left) and *STAT3* (right) expression levels and normalizing to *TBP* expression; shown are means ± SEM (*n* = 6). **D.** *In vivo* treatments using CpG-*RELA*siRNA or CpG-*STAT3*siRNA reduce clonogenic potential of PC3 prostate cancer cells. Tumor cells isolated from NSG mice treated as indicated were used for colony-forming assays; shown are mean numbers of colonies ± SEM (*n* = 5).

While targeting stem cell-like cells could overcome therapeutic resistance of late-stage cancers, the task remains challenging [21]. TLR9 expression in prostate cancer propagating cells creates an opportunity to use this receptor as a target for the delivery of therapeutic molecules, such as CpG-siRNA [28]. As we previously demonstrated, siRNAs linked to TLR9 ligands (e.g. CpG ODNs) are internalized by hematopoietic cells, followed by the TLR9-mediated cytoplasmic siRNA release and target gene silencing [28]. We verified that in the absence of any transfection reagents *in vitro*, TLR9-positive PC3-luc and DU145 prostate cancer cells, efficiently internalize CpG-siRNA (Figure 4A and Supplementary Figure S7A). Next, we evaluated the impact of silencing *NF-κB/RELA* or *STAT3* on the growth of PC3-luc and DU145 tumors. The intratumoral injections of CpG-*RELA*siRNA alone, CpG-*STAT3*siRNA alone or combination thereof, but not CpG-scrambled RNA, induced target gene silencing and inhibited growth of PC3 tumors. The observed therapeutic effects were more pronounced for targeting *RELA* rather than *STAT3* or both transcription factors together. In contrast, similar experiments using DU145 tumors showed comparable growth inhibitory effects of both CpG-*RELA*siRNA and CpG-*STAT3*siRNA (Figure 4B, right; Supplementary Figure S7B). These results may reflect various degree of STAT3-dependency in both tumor models as well as less effective silencing of STAT3 compared to RELA in target cells (Figure 3C). Importantly, the colony-forming assays confirmed that intratumoral injections of CpG-*RELA*siRNA and CpG-*STAT3*siRNA reduced clonogenic potential of tumor cells (Figure 4D). Consistently with our earlier studies, we did not observe target gene silencing or any antitumor effects of CpG-*STAT3*siRNA in TLR9-negative tumor xenotransplants in immunodeficient mice (Supplementary Figure S8) [28]. Our ongoing studies using syngeneic prostate cancer models should reveal the complete therapeutic potential of immunostimulatory CpG-siRNAs.

Previous *in vitro* studies suggested that TLR9 promotes prostate cancer cell proliferation and/or invasiveness [7]. However, to our knowledge this is the first report of TLR9′s role in promoting prostate cancer cells' self-renewal and tumor-propagating potential. This is a result of the concerted expression of genes related to cancer stem cell maintenance through NF-κB/RELA and STAT3 as downstream mediators of TLR9 signaling. Finally, we demonstrate the feasibility to use TLR9-mediated siRNA delivery for targeting prostate cancer-propagating cells *in vivo*, thereby halting tumor progression. Targeting of TLR9^+^ tumor-propagating cells alone or in combination with antiproliferative agents has potential to address an unmet need for treatment of patients with advanced and poorly differentiated prostate cancers.

## DISCUSSION

Previous studies reported that prostate cancer cells express innate immune receptors, such as TLR9, normally restricted to the hematopoietic cell lineage [2, 5, 7]. Rather than becoming immunogenic, TLR9^+^ prostate cancers were less differentiated, more aggressive and prone to reoccur [8, 29]. Our study reconciles these observations as it identifies a novel function of TLR9 signaling in cancer cells and underscores the role of inflammation in prostate cancer progression. We demonstrate for the first time that TLR9 expression promotes tumor-propagating potential of prostate cancer cells *in vivo*. The TLR9^+^ prostate cancer cells have increased both clonogenic potential and the ability to differentiate into osteoblast- or adipocyte-like cells similarly as bone marrow mesenchymal stem cells. These phenotypic changes are associated with concerted expression of tumorigenic mediators, including a set of genes related to cancer stem cell maintenance and tumor-propagating potential. We identify NF-κB/RELA and STAT3 as two critical downstream mediators of TLR9 signaling. Both NF-κB/RELA and STAT3 coordinate transcription *NKX3.1* and *KLF4* stem cell-related genes. Finally, silencing of *RELA* or *STAT3* specifically in TLR9^+^ tumor-propagating cells inhibits growth of established prostate cancers *in vivo*.

Our results align with earlier reports demonstrating elevated TLR9 expression in prostate cancers with higher Gleason scores and in the cell lines established from more advanced tumor stages such as PC3 or DU145 [5, 7, 29]. Previous *in vitro* studies suggested that TLR9 activity promotes cancer cell proliferation [30] and/or invasiveness [7]. However, to our knowledge this is the first report of TLR9's role in promoting prostate cancer cells' self-renewal and tumor-propagating potential. Several studies suggested the existence of undifferentiated population of prostate cancer stem cells with self-renewing potential, which enable serial tumor transplantation in immunodeficient mice [20, 21, 31]. Growing evidence also links prostate cancer stem cells to castration resistance and tumor recurrence [20, 32]. We found that TLR9 expression is essential for prostate cancer cells ability to differentiate into adipocytes or osteoblasts [22]. Given the similarity between prostate cancer cells and bone marrow mesenchymal stem cells, TLR9^+^ tumor-propagating cells may play a role in the metastatic tumor spread to bone. This possibility will be explored in our further studies. Noteworthy, recent studies suggested that non-stem cancer cells can dedifferentiate into tumorigenic cells [33]. The notion of stem cell phenotype reversibility find some support in our findings that forced expression or silencing of TLR9 can affect tumor-propagating potential of prostate cancer cells. As recently demonstrated, TLR9 expression in cancer cells is upregulated in response to genotoxic stress caused by irradiation or chemotherapy [6]. Therefore, expansion of cancer stem cell population could result from TLR9-mediated response to environmental conditions, thereby limiting the outcome of cytotoxic tumor therapies. In fact, sterile tissue injury or tumor irradiation can stimulate TLR9 signaling *in vivo* through release of natural TLR9 agonists such as mitochondrial DNA [9, 10, 34].

Downstream TLR9 signaling involves NF-κB activation and indirectly induces STAT3 signaling. [4, 9, 11] In our studies, TLR9 expression upregulated several well known activators of tumorigenic STAT3 signaling in prostate cancer cells, such as EGFR, IL-6 and VEGF [12]. We demonstrate that TLR9/NF-κB/STAT3 signaling axis operates in prostate cancer cells to promote expression of tumorigenic and stem cell-related genes. While both NF-κB and STAT3 are well known mediators of prostate carcinogenesis and progression, their collaboration in promoting prostate cancer-propagating cells has not yet been shown [15, 35]. As suggested by both our transcriptome and protein analyses, the link between TLR9/NF-κB and STAT3 signaling likely relies on IL-6 secretion by cancer cells. IL-6 is known for inducing rapid STAT3 phosphorylation through the IL-6R-associated Janus family kinases (JAKs) [36]. IL-6-mediated activation of STAT3 in human prostate cancers is well documented and related to androgen-independence [17, 37]. As recently suggested, IL-6/STAT3 signaling also promotes stem cell-like phenotype of cancer cells [38]. We cannot exclude the contribution of other signaling pathways to transcriptional activity induced by TLR9. In fact, based on our *in vitro* experiments TLR9-triggered gene expression is sensitive not only to inhibitors of NF-κB and STAT3, but also to Src and PI3K/Akt inhibitors (Moreira unpublished data). Recent reports demonstrated synergism between IL-6/STAT3 and PI3K/Akt or Src pathways in promoting prostate cancer aggressiveness and progression [37, 39]. Our findings underscore the possibility that these signaling pathways converge to orchestrate functionally overlapping set of genes. As shown by this study, TLR9/NF-κB/STAT3 signaling coordinates expression of genes related to differentiation and renewal of normal or cancer stem cells. Several of the identified targets genes are known mediators of prostate cancer cell “stemness” and self-renewal, such as *NKx3.1* [24], *BMI-1* [23], and *SOX-4* [25]; some are also embryonic stem cell (ESC) regulators, such as *KLF4* and *SOX2* [40]. However, TLR9 signaling is not required for expression of the two canonical ESC transcription factors *NANOG* and *OCT4*. This is in contrast to recently shown direct TLR4/NANOG signaling in liver cancer-initiating cells [41], which may indicate diversity of molecular mechanisms regulating stem cell phenotype in various solid tumors.

While targeting stem cell-like cells gained interest as a potential method to overcome therapeutic resistance of late-stage cancers, such task remains challenging [21, 31]. TLR9 expression in prostate cancer cells creates an opportunity to use this receptor as a target for the delivery of therapeutic molecules, such as CpG-siRNA conjugates [42]. Doses and uptake of CpG-siRNA by TLR9^+^ human prostate cancer cells were comparable to our previous observations in blood cancer models [28]. The proof-of-principle experiments using CpG-siRNAs to target tumorigenic NF-κB/RELA and STAT3 signaling in xenotransplanted TLR9^+^ prostate tumors confirmed therapeutic efficacy of such strategy. Targeting either of the two tumorigenic transcription factors inhibited tumor growth and reduced clonogenic potential of tumor cells. Overall, our findings suggest that TLR9-mediated siRNA delivery targets prostate cancer-propagating cells, thereby halting tumor progression. Further *in vivo* studies will elucidate effects of inhibiting TLR9-mediated signaling, which should trigger differentiation of cancer-propagating cells into tumor cells with potentially greater sensitivity to standard therapies [43]. Therefore, targeting TLR9^+^ tumor-propagating cells alone or in combination with expanding panel of antiproliferative agents, can address an unmet need for treatment of patients with advanced and poorly differentiated prostate cancers.

## MATERIALS AND METHODS

### Pathological analysis

Investigation has been conducted in accordance with the ethical standards and according to the Declaration of Helsinki and according to national and international guidelines and has been approved by the authors' institutional review board. Prostate cancer specimens from patients' samples were acquired from the Biospecimen Repository (COH) with the prior Institutional Review Board approvals (IRB09213), with written, informed consent of all patients. 4 μm sections prepared from the selected 48 prostate cancer specimens were deparafiinized, stained using human TLR9-specific antibodies (IMG305a; Imgenex) and evaluated by a pathologist (COH).

### Cell lines

Human prostate cancer cells: DU145, PC3 expressing luciferase and STAT3 (PC3-luc; Supplementary Figure S7C), LNCaP-S17 (LN-S17) stably expressing IL-6 [19] were kindly provided by Dr. Richard Jove (Vaccine&Gene Therapy Institute, FL), while LNCaP were purchased from ATCC. All cells were authenticated and clear from contamination by other cell types (Biosynthesis, Lewisville, TX). The PC-TLR9^LO^ and PC-TLR9^HI^ cells were generated using lentiviral transduction of pKLO-puro.1-shTLR9 or control pKLO-puro.1 vectors (Sigma-Aldrich) into PC3-luc cells, respectively, followed by selection of stable cell lines in puromycin (1.5 μg/ml). The sequence of shTLR9 was: 5′-CCGGCCACTTCTATAACCGGAACTTCTCGAGAAG TTCCGGTTATAGAACTGGTTTTTG-3′. To generate the LNCaP-TLR9^+^ and LN-TLR9^HI^ cells, the full length human TLR9 cDNA (Addgene) was cloned into pLVX-EF1α vector and lentivirally transduced into LNCaP and LN-S17 cells to select stable clones.

### *In vitro* spheroid and clonogenic assays

For the spheroid formation assays, 24-well culture dishes coated with 0.6%(w/v) agarose were overlayed using 1 × 10^4^ of freshly isolated tumor cells suspended in the agarose-medium mixture (RMPI/10%(v/v)FCS/0.3%(w/v) agarose). The colonies were counterstained and counted after 1–2 weeks of culture. The clonogenic assay design was reported by others [27].

### *In vivo* experiments

NOD/SCID/IL-2RγKO mice (NSG), aged 6–8 weeks, were purchased from the Jackson Laboratory. Mouse care and experimental procedures were performed under pathogen-free conditions following protocols approved by the Institutional Animal Care and Use Committee (COH). The limited-dilution assays to calculate tumor-propagating cell frequency were performed as described elsewhere using the ELDA software to calculate frequencies and probability estimates [21]. The CpG-siRNA design was described previously [28]. The sequence of the CpG-*RELA*siRNA (deoxyribonucleotides are underlined; asterisks indicate phosphothioation sites): CpG-siRNA(antisense strand): 5′ G*G*TGCATCGATGCAGG*G*G*G*G-Linker-UCC UUUUACGUUUCUCCUCAAUCCGGU 3′ siRNA(sense strand): 5′ CGGAUUGGAGAAACGUAAAAGGA 3′.

### Western blotting

Total cellular lysates were prepared as previously reported [28] and analyzed using antibodies specific to TLR9 (Imgenex), tyrosine-phosphorylated STAT3, total STAT3, BMI-1, COL1A1, KLF4, NKX3.1, SOX-4 (Santa Cruz) and β-actin (Sigma).

### Quantitative real-time PCR

The RNA extraction, reverse transcriptase and real time PCR reactions were performed using CFX96 Real-Time PCR Detection System (Bio-Rad) and the specific pairs of primers designed for *BMI-1, COL1A1, KLF4, NANOG, NKX3.1, OCT-4, SOX2* and *SOX4* (sequences provided in the Supplementary Materials) as previous described [28]. The data were normalized to the *TBP* levels and the relative gene expression levels were calculated using the 2^−ΔΔCt^ method.

### Flow cytometry

For uptake studies, following the incubation with fluorescently-labeled oligonucleotides cells were analyzed using C6 flow cytometer (BD Biosciences) and analyzed using FlowJo software (Tree Star).

### Chromatin Immunoprecipitation (ChIP) assays

ChIP assays were carried out with a EipTect ChIP OneDay (Qiagen) following the manufacturer's instructions. For ChIP-re-ChIP assay, the immunoprecipitates were subjected to a second round of immunoprecipitation. The presence of specific DNA fragments was quantified using qPCR and specific primers listed in the Supplementary Methods.

### Statistics

Unpaired *t* test was used to calculate two-tailed *P* value to estimate statistical significance of differences between two treatment groups. One- or two-way ANOVA plus Bonfeerroni post-test were applied to assess differences between multiple groups or in tumor growth kinetics experiments, respectively. Statistically significant *P* values were indicated in figures as follows: ***, *P* < 0.001; **, *P* < 0.01 and *, *P* < 0.05. Data were analyzed using Prism software v. 6.01 (GraphPad).

## SUPPLEMENTAL MATERIALS AND METHODS TABLE AND FIGURES


