# Supplementary material for: TLR9 signaling through NF-κB/RELA and STAT3 promotes tumor-propagating potential of prostate cancer cells
Source: Oncotarget. 2015 May 22;6(19):17302–13. doi: 10.18632/oncotarget.4029 (PMC4627309; doi:10.18632/oncotarget.4029)
Supplement: Supplementary file 1 [file oncotarget-06-17302-s001.pdf]

SUPPLEMENTAL MATERIALS AND METHODS

Global gene expression analysis

Total RNA samples extracted from xenotransplanted PC-TLR9^{LO} and PC-TLR9^{HI} tumors using mirVana™ Isolation Kit (Ambion) were sequenced on Illumina HiSeq2000 generating 40bp long reads. The sequences that passed the default chastity filter were aligned with the mouse reference genome (Genome Browser, UCSC, CA) using the open source RNA-Seq alignment TopHat software (v.1.3.1) to identify differential gene expression. The results were converted to reads/kilo base of total exon length/million mapped (RPKM) reads using GenomicsSuite v.6.12.0713 (Partek) and normalized to gene models in the NCBI RefSeq database with a stringent cutoff of 0.1 RPKM and the false discovery rate (FDR) < 0.05. Differentially expressed mRNA had fold change cutoff of 1.5 and the *P*-value with FDR cutoff of 0.05. The expression profiling data were submitted to the Gene Expression Omnibus (GSE45180). The biological functions of the differentially expressed mRNAs were predicted using the Ingenuity Pathway Analysis (Qiagen).

In vitro clonogenic and differentiation assays

The clonogenic assay design was reported by others [27]. Briefly, we cultured 10^3 of viable single cells for 6–12 days to evaluate microscopically the formation of holo- and para-clone colonies. The osteoblastic and adipogenic differentiation assays were performed as described elsewhere [22].

Uptake studies

The intracellular localization of the fluorescently labeled CpG-*STAT3*siRNA^{Cy3} (500 nM) was assessed using confocal time-lapse microscopy in living PC3 cells. Cells were cultured on 35/14 mm #1.5 glass bottom tissue dishes (MetTek, Ashland, MA). Images were acquired using C-Apochromat 40x/1.2 water immersed objective and LSM software (Zeiss), in 20–30 min time increments, in Z stacks. Microscope chamber with environmental control (37°C/5% CO_2) was used in all experiments with living cells.

Chromatin Immunoprecipitation (ChIP) Assays

The presence of specific DNA fragmentswas quantified using qPCR and specific sets of primers listed below.

Gene target		Sequence	#UPL
BMI-1	Forward	5′-gtgactctgggagtgacaagg-3′	18
	Reverse	5′-ggcaaacaagaagaggtgga-3′	
COL1A1	Forward	5′-gggattccctggacctaaag-3′	67
	Reverse	5′-ggaacacctcgctctcca-3′	
FBN1	Forward	5′-cctggttacttccgcatagg-3′	21
	Reverse	5′-tcatccatttcaccactgaca-3′	
NANOG	Forward	5′-cagctgtgtgtactcaatgatagattt-3′	67
	Reverse	5′-tctggaaccaggtcttcacc-3′	
KLF-4	Forward	5′-gcgagtctgacatggctgt-3′	32
	Reverse	5′-gtcgcttcatgtgggagag-3′	
OCT-4	Forward	5′-tttttctttgagacagagtcttgc-3′	19
	Reverse	5′-gctgagatcacgccactg-3′	
SOX-2	Forward	5′-atgggttcggtggtcaagt-3′	19
	Reverse	5′-ggaggaagaggtaaccacagg-3′	
NKx3.1	Forward	5′-agaacgaccagctgagcac-3′	72

(*Continued*)

Gene target		Sequence	#UPL
	Reverse	5′-tccaacagataagaccccaag-3′	
SOX-4	Forward	5′-cggtgagagagcgagagaga-3′	19
	Reverse	5′-gcctctcgcagaggagttatag-3′	
STAT3	Forward	5′-ctgcctagatcggctagaaaac-3′	25
	Reverse	5′-ccctttgtaggaaactttttgc-3′	
RELA	Forward	5′-tcatgaagaagagtcctttca-3′	39
	Reverse	5′-ctggcttggggacagaag-3′	
NKX3.1 Chip	Forward	5′-tggcaaaggtgttttccttc-3′	71
	Reverse	5′-gacctcaaagaggcatgacaa-3′	
KLF-4 Chip	Forward	5′-gcgagtctgacatggctgt-3′	
	Reverse	5′-cggagcgatactcacgttattc-3′	

Supplementary Table S1. TLR9 expression levels in 48 primary human prostate cancer

	TLR9 score		
**Gleason grade**	**+**	**++**	**+++**
**3+3**	4	6	2
**3+4**	2	8	4
**4+3**	-	6	7
**4+4**	-	3	4
**4+5**	-	-	2

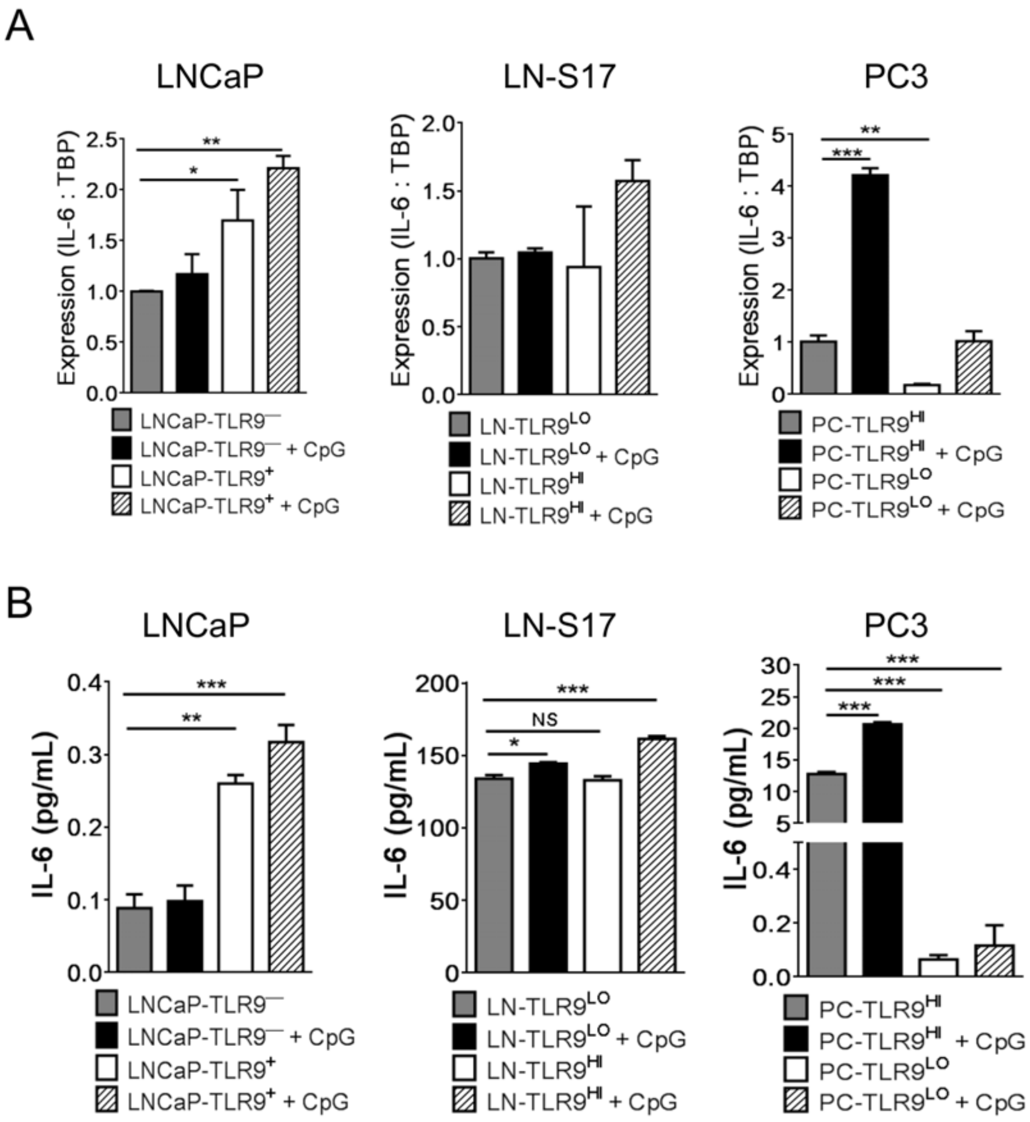

Supplementary Figure S1: IL-6 expression correlates with TLR9 levels in prostate cancer cells. The levels of IL-6 mRNA **A.** and protein **B.** were assessed in LNCaP, LN-S17 and PC3 cell variants, using qPCR and ELISA, respectively. Cells were treated with or without CpG ODN (1 µg/ml) for 48 hours. The IL-6 mRNA levels were normalized to *TBP* expression. The IL-6 protein was measured in supernatants. Shown are means ± SEM from three independent experiments.

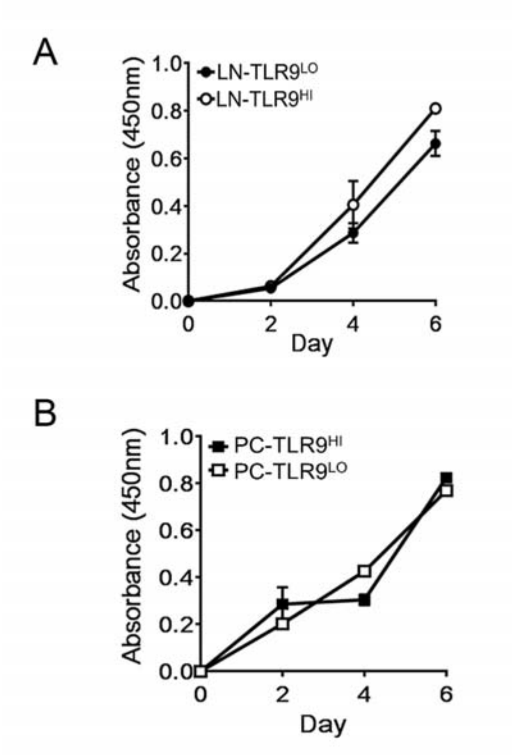

Supplementary Figure S2: TLR9 expression levels do not affect proliferation of prostate cancer cells *in vitro*. Changes in TLR9 expression do not affect proliferation of LNS17 **A.** and PC3 **B.** cell variants. Cells were cultured in complete media and growth was assessed using XTT assays at day 2, 4 and 6; means ± SEM from three independent experiments.

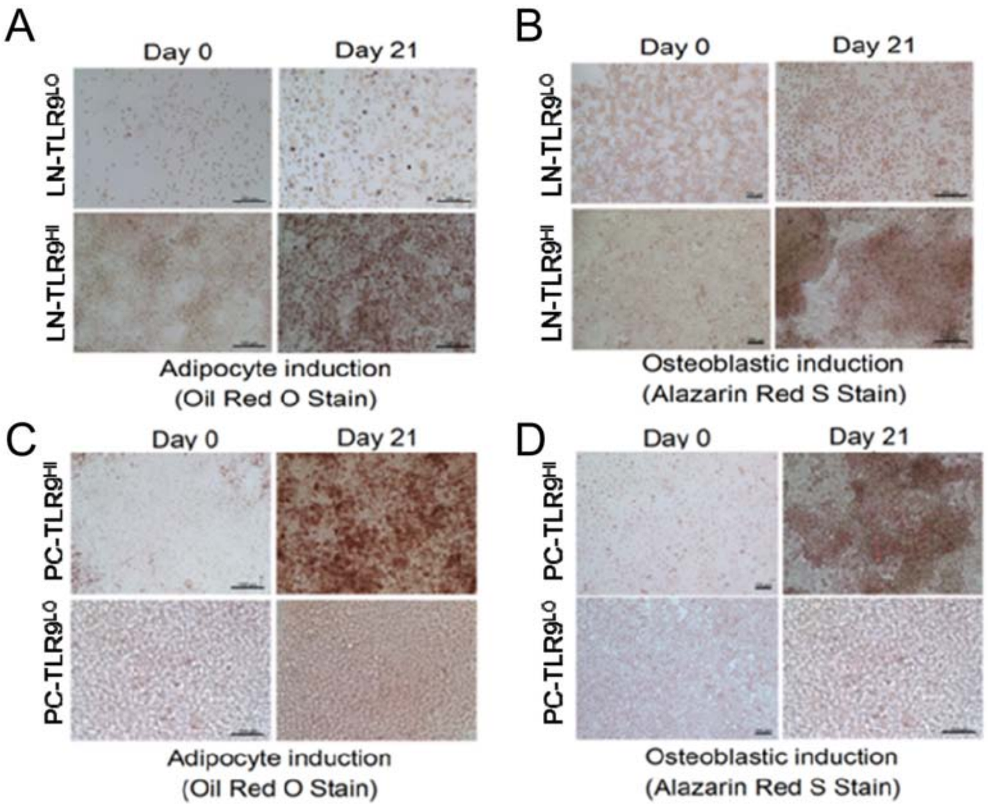

Supplementary Figure S3: Adipogenic and osteoblastic differentiation of prostate cancer cells requires TLR9 expression. LNS17 **A, B.** or PC3 **C, D.** cell variants were cultured for 21 days under adipogenic (A, C) or osteoblastic (B, D) induction conditions for 21 d. Cells were stained using Oil Red O for triglycerides/lipids or using Alazarin Red S for detection of calcium deposits. Shown are representative results from two independent experiments.

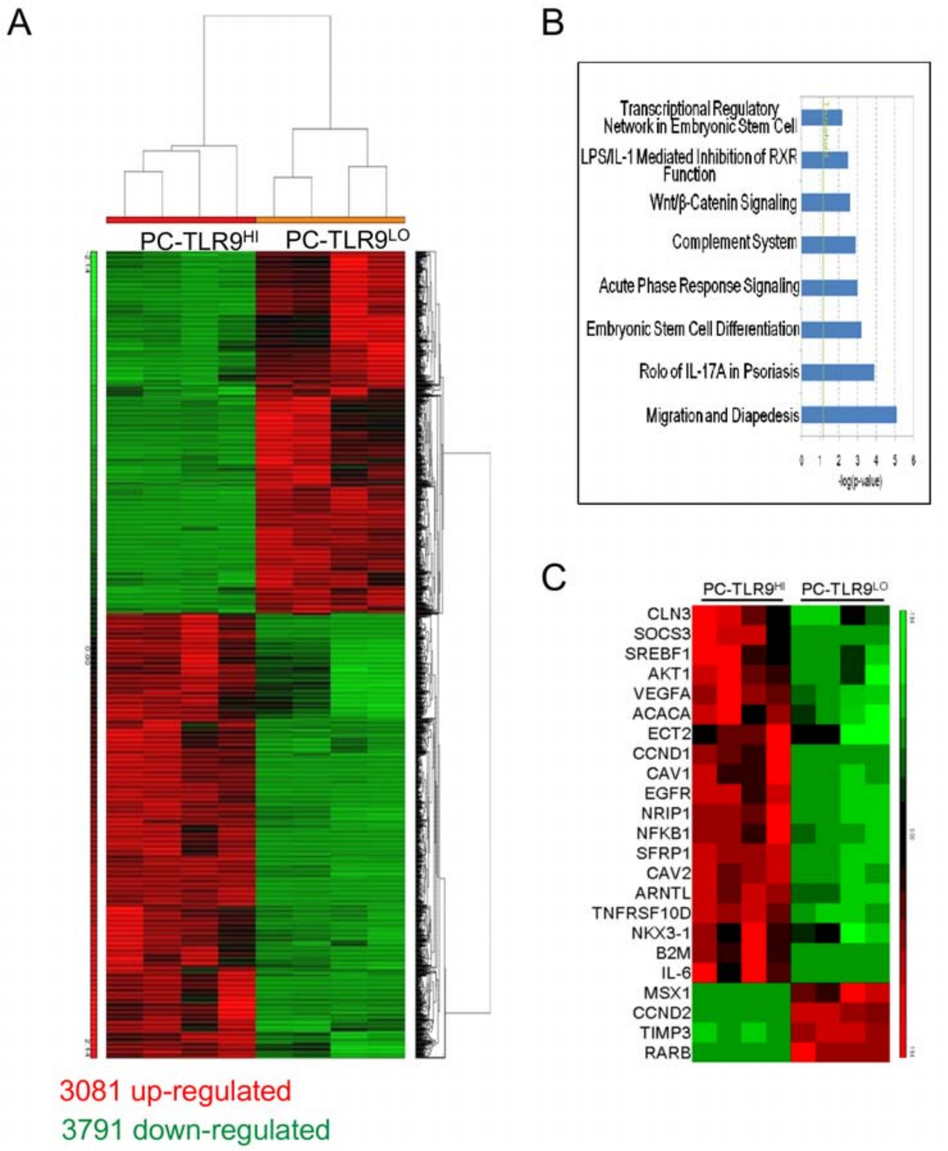

Supplementary Figure S4: TLR9 orchestrates expression of tumorigenic and stem cell-related genes. A. Overview of the global gene expression pattern in PC-TLR9^{HI} compared to PC-TLR9^{LO} tumors as assessed by RNAseq and the hierarchical clustering analysis. Each column shows the expression levels for RNA samples derived from a single tumor from both groups. **B.** Top functional categories affected by TLR9 silencing in PC3 tumors. The list of genes differentially expressed was analyzed using IPA analysis. **C.** Heatmap showing selected differentially expressed genes related to prostate cancer growth, survival, progression and stem cell maintenance. High and low gene expression was indicated by red or green color, respectively.

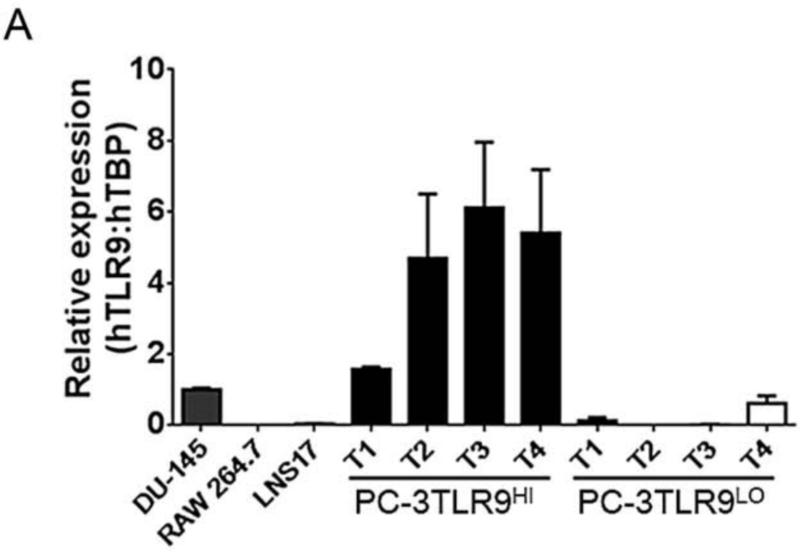

B

Gene Symbol	Fold Regulation	p-value
ACACA	-6,26	0,00351
AKT1	-5,04	0,001098
ARNTL	-10,98	0,000907
CAV1	-22,74	0,002242
CAV2	-12,45	0,000014
CCND1	-27,11	0,000234
CLN3	-7,75	0,011407
ECT2	-8,68	0,000007
EGFR	-37,72	0,004222
IL6	-38,35	0,002515
NFKB1	-9,08	0,000049
NKX3-1	-5,31	0,002497
NRIP1	-24,95	0,000016
SFRP1	-17,55	0,000702
SOCS3	-14,82	0,008796
SREBF1	-8,97	0,023082
TNFRSF10D	-9,40	0,000139
VEGFA	-11,40	0,000105
B2M	-17,54	0,000017
CCND2	67,61	0,002259
MSX1	12,24	0,000265
RARB	4,40	0,000168
TIMP3	23,41	0,000041

C

Upstream regulator	Activation z-score	p-value of ovelap
Inhibited		
STAT3	-2.627	1.90E-07
HNF1A	-3.214	1.00E-06
KMT2D	-2.223	3.90E-06
TGIF1	-2.265	1.53E-05
NFkB (complex)	-4.553	4.57E-05
NR113	-3.501	5.81E-05
CPBA	-3.011	8.08E-05
RELA	-3.849	1.32E-04
ZNF217	-2.121	1.34E-04
STAT2	-2.646	7.05E-04
REST	-2.395	1.05E-02
CEBPB	-2.498	2.23E-03
STAT1	-3.362	6.15E-03
NFE2L2	-2.567	6.17E-03
FOXL2	-2.425	6.94E-03
COMMD3-BMI1	-2.219	7.41E-03
HMGB1	-2.387	2.55E-02
NR112	-2.078	1.27E-01
IRF5	-2.143	1.38E-01
IRF7	-2.741	3.22E-01
Activated		
MEOX2	2.534	2.32E-06
NEUROG3	3.411	1.11E-05
KMT2A	2.173	1.95E-04
PRDM1	2.272	3.48E-04
NEUROD1	2.437	4.17E-04
NKX2-5	2.219	7.05E-04
PITX2	2.166	1.18E-03
GFI1	2	7.65E-03
HLX	2.219	9.69E-03
SIRT1	2.606	1.86E-02

Supplementary Figure S5: Validation of the RNAseq results using qPCR arrays to assess expression of prostate cancer related genes in PC3 tumors variants. A. The *TLR9* expression was assessed using qPCR in total RNA samples isolated from viable cancer cells freshly derived from *in vivo* grown of PC3 tumor variants. Cultured LNS17, DU-145 and RAW 264.7 cells were used as controls; means ± SEM ($n = 3$). **B.** Validation of RNAseq results using prostate cancer-specifc qPCR Array (Qiagen). **C.** List of upstream regulators affected by *TLR9* silencing in PC3 tumors predict using IPA.

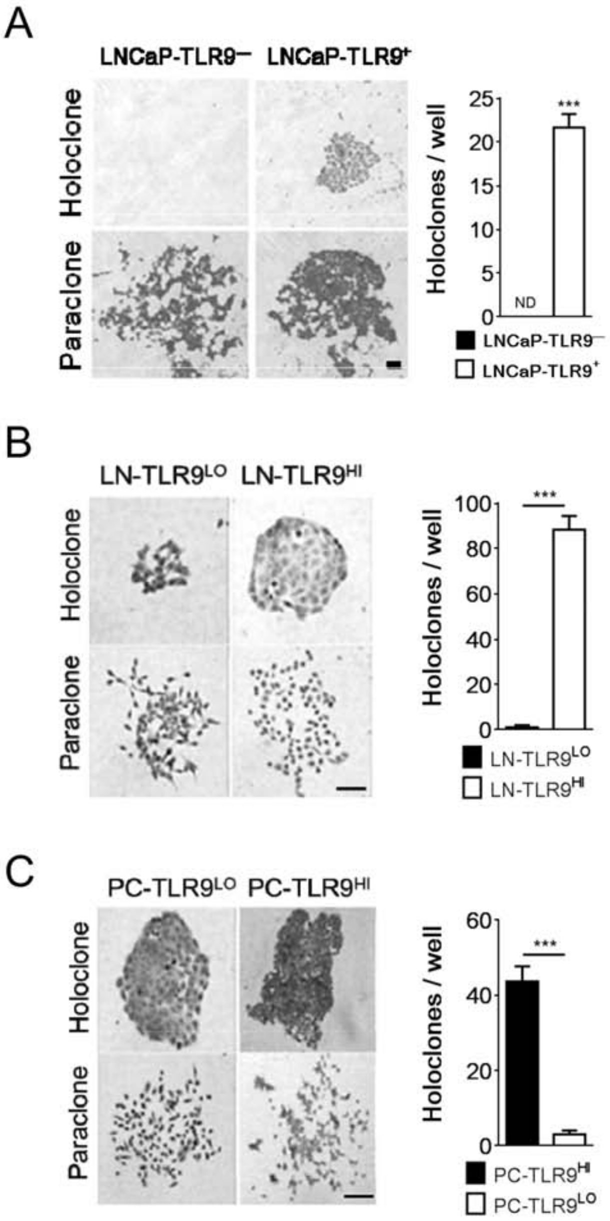

Supplementary Figure S6: TLR9 levels correlate with the ability of prostate cancer cells to form holoclone colonies *in vitro*. Representative phase contrast images (left panels) showing paraclone and holoclone formation by cultured LNCaP **A.**, LNCaP-S17 **B.** and PC3 **C.** variants and holoclone counts (right panels); means ± SEM from three independent experiments. Scale bar = 100 µm.

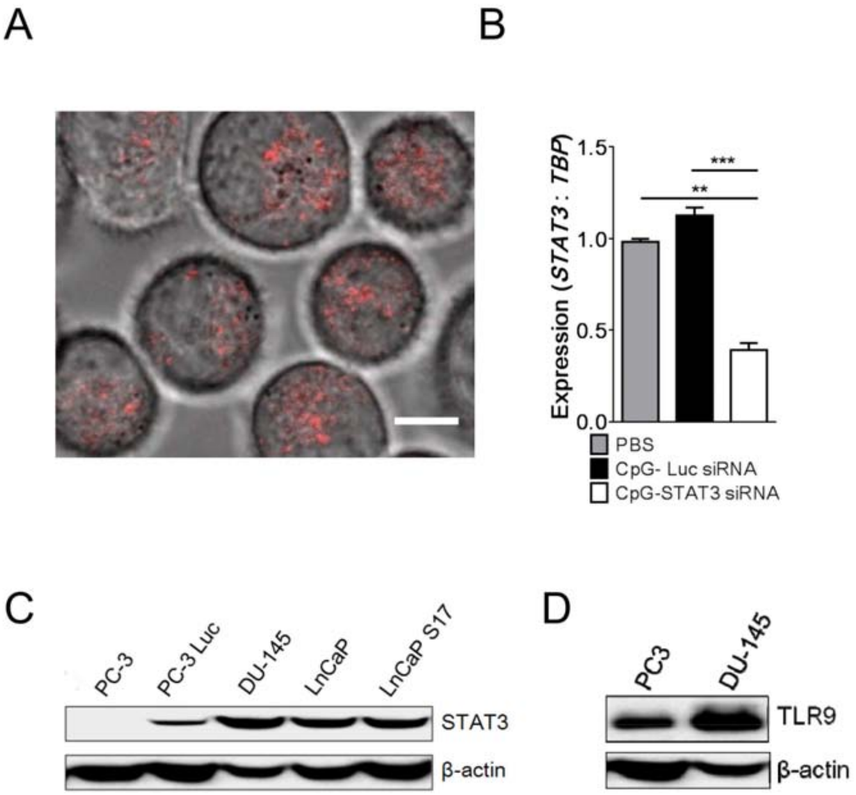

Supplementary Figure S7: CpG-*STAT3*siRNA uptake *in vitro* and target gene silencing in DU145 prostate tumors *in vivo*. A. The intracellular localization of the CpG-siRNA^{Cy3} (500 nM/1 h) was assessed using confocal time-lapse microscopy in living PC3 cells; scale bar = 100 µm. **B.** The silencing effect of CpG-*STAT3*siRNA in experiment described in the main Figure 4B was verified using qPCR to measure and *STAT3* **B.** expression levels and normalizing to *TBP* expression; shown are means ± SEM (n = 5). **C.** STAT3 expression is common in various prostate cancer cells and their sublines except for parental PC3 cells. Shown are representative results from one of three independent Western blot analyses detecting total STAT3 protein levels in relationship to β-actin used as a loading control. **D.** PC3-and DU-145 cells express high levels of TLR9. Shown are representative results of western blot analysis from one of three independent experiments.

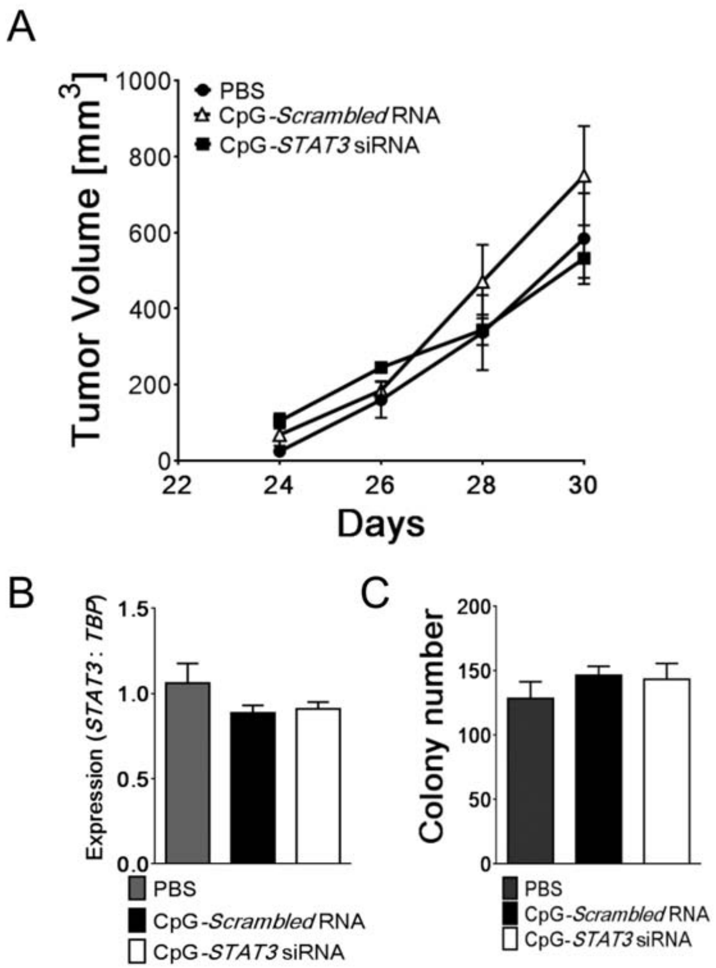

Supplementary Figure S8: CpG-*STAT3* siRNA fails to inhibit growth of TLR9-negative prostate tumors *in vivo*. A. NSG mice with established PC3-TLR9^{LO} tumors were treated at day 24 after implantation using IT injections of CpG-*STAT3*siRNA, CpG-scrambled RNA (5 mg/kg) or PBS every other day while measuring tumor volumes. **B.** Lack of the *STAT3* silencing in PC3-TLR9^{LO} prostate cancer cells as measured using qPCR; shown are means ± SEM (n = 6). **C.** The clonogenic potential of PC3-TLR9^{LO} cells is not affected by CpG-*STAT3*siRNA treatments. Tumor cells isolated from NSG mice treated as indicated were used for colony-forming assays; shown are mean numbers of colonies ± SEM (n = 5).
